# Kidney Outcomes in Structural Heart and Cardiac Surgical Trials: The Case for Longitudinal Endpoint Reporting

**DOI:** 10.7759/cureus.116741

**Published:** 2026-09-22

**Authors:** Achille Peiris, Diego de Silva

**Affiliations:** 1 Nephrology, Balboa Nephrology Medical Group, San Diego, USA

**Keywords:** cardiac surgery outcomes, cardiorenal syndrome, cardiovascular-kidney-metabolic syndrome, chronic kidney disease progression, clinical trial endpoints, estimated glomerular filtration rate, transcatheter aortic valve replacement, valve academic research consortium

## Abstract

The 2026 American Heart Association/American College of Cardiology/American Diabetes Association/American Society of Nephrology guideline on cardiovascular-kidney-metabolic syndrome establishes kidney function as a determinant of cardiovascular risk rather than an adjacent comorbidity. Endpoint architecture in structural heart and cardiac surgical research has not been revised accordingly. Valve Academic Research Consortium 3 standardized acute kidney injury reporting with considerable rigor, staging injury on Kidney Disease: Improving Global Outcomes serum creatinine criteria and requiring that new renal replacement therapy be reported discretely, but its scope is confined to the periprocedural window. No endpoint tracks filtration trajectory across follow-up, no threshold within the standard defines chronic kidney disease stage progression, and initiation of maintenance dialysis years after intervention has no reporting convention distinct from temporary replacement therapy in the immediate postoperative period. Pivotal trials consequently adjudicate cardiovascular outcomes for five years and kidney outcomes for 30 days. Registry evidence suggests the omission is consequential, with rapid filtration decline documented in more than a third of patients carrying preexisting kidney impairment within five years of cardiac surgery. This editorial argues for longitudinal reporting of estimated glomerular filtration rate, prespecification of stage progression and maintenance dialysis as adjudicated endpoints, and nephrology representation on trial steering committees with a defined role in kidney endpoint specification.

## Editorial

The 2026 American Heart Association/American College of Cardiology/American Diabetes Association/American Society of Nephrology guideline on cardiovascular-kidney-metabolic syndrome formalizes a clinical intuition that long preceded it, namely that metabolic derangement, declining filtration, and structural heart disease are expressions of a shared pathophysiology rather than coincident diagnoses [1]. Four societies drafted it jointly, and kidney function appears in it as a determinant of cardiovascular risk rather than as a comorbidity recorded in the margin.

Endpoint architecture in structural heart and cardiac surgical research has not been revised to match. Patients with chronic kidney disease enroll in these trials in substantial numbers, and their creatinine is drawn at nearly every protocol visit for years. What the literature ultimately reports about them concerns the first 30 days.

The standard read against its own remit

The Valve Academic Research Consortium 3 (VARC-3) is a rigorous document and deserves to be assessed against what it set out to do. Acute kidney injury sits among 15 defined clinical endpoints, staged on Kidney Disease: Improving Global Outcomes serum creatinine criteria, with urine output thresholds reserved for investigations dedicated to kidney injury itself [2,3]. VARC-3 went further, requiring that new renal replacement therapy, temporary or permanent, be reported discretely as stage 4, and that patients established on chronic dialysis be removed from the denominator [2]. These are not incidental refinements. They reflect close attention to how kidney injury actually behaves in the periprocedural window.

The limits of the construct

What constrains the standard is its time horizon rather than any imprecision in its definitions. No endpoint tracks the trajectory of kidney function across the observation period. Filtration slope goes unreported. Stage progression carries no threshold defined within the standard. A patient who initiates maintenance dialysis in the third year is a categorically different patient from one requiring temporary replacement therapy on the second postoperative day, though summary reporting collapses the distinction with some regularity. VARC-3 itself notes that estimated glomerular filtration rate is the measure clinicians rely on to classify severity, then defines acute injury on creatinine, which is the correct choice for its purpose [2]. No longitudinal analogue was specified, because longitudinal kidney function fell outside the scope the consortium had claimed.

The pivotal literature bears out the consequence. At five years, the PARTNER 3 investigators reported mortality, stroke, rehospitalization, valve hemodynamics, bioprosthetic valve failure, and health status, each adjudicated across the full interval with evident rigor [4]. Kidney trajectory is absent from the longitudinal outcome set. The trial answered its prespecified questions faithfully; this was not among them.

What measurement reveals

Such evidence as exists on kidney trajectory after cardiac intervention originates outside the randomized literature, and it does not console. A retrospective multicenter cohort of 27,483 adults undergoing cardiac surgery identified 3,512 (12.8%) patients with preexisting chronic kidney disease at stages G3a through G5. Over a median follow-up of seven years, with competing risk analysis accounting for mortality, five-year cumulative incidence reached 38.7% for rapid progression, defined as confirmed decline in estimated glomerular filtration rate of at least 5 mL/minute/1.73 m² annually. Stage progression, defined as a confirmed decline of at least 25% in estimated glomerular filtration rate accompanied by advancement in chronic kidney disease stage, occurred in 23.8%, and kidney failure in 5.5% [5].

More than a third of patients who entered surgery with impaired filtration sustained rapid functional loss within five years. That figures of this magnitude issue from a registry rather than from the randomized programs that established these therapies state the problem in compressed form: observational designs have taken up ground randomization was built to hold, and they carry the confounding randomization exists to dissolve.

The cost is borne in a conversation trialists rarely witness. Asked whether a patient at stage 4 should proceed to transcatheter or surgical replacement, the nephrologist finds the cardiovascular literature capable of supporting a confident recommendation and wholly silent on where that patient’s filtration will sit 18 months later under either strategy.

Provenance of the omission

Endpoint hierarchies are authored by whichever specialty owns the intervention, and they bear the impress of that vantage. Kidney outcomes entered valve trial frameworks classified as procedural safety events because that is how a proceduralist encounters them, in the catheterization laboratory or the intensive care unit, within hours. Steering committees are constituted from interventionalists, surgeons, imagers, and statisticians; nephrologists are consulted on eligibility thresholds and thereafter, as a matter of custom, not consulted again. The resulting evidence base is calibrated to one organ system in a cohort enrolled partly because two are compromised.

The cardiovascular-kidney-metabolic framework makes that arrangement difficult to sustain on its own logic. A trial adjudicating cardiac outcomes across five years and kidney outcomes across 30 days has characterized one face of the syndrome and reported it as the whole.

Remediation

Report estimated glomerular filtration rate as a longitudinal outcome at the intervals already established for cardiovascular endpoints, using a single estimating equation across arms. The values reside in the study record. Publishing them is an analytic decision rather than a logistical one.

Prespecify and adjudicate two additional outcomes over follow-up, chronic kidney disease stage progression and initiation of maintenance dialysis, both distinct from the periprocedural stage 4 event VARC-3 already defines. Ascertainment for each is unambiguous, and the consequence to the patient is considerable.

Seat a nephrologist on the steering committee of any trial enrolling substantial numbers of participants with kidney disease, vested with a defined role in how kidney endpoints are specified. Consultation on eligibility thresholds and participation in design are different undertakings, and the distinction is legible in what eventually appears in print.

Conclusions

None of this indicts the trials that established these therapies, nor the consortium that brought coherence to their reporting. Both accomplished what they undertook, and patients have benefited accordingly. The claim is narrower. The population enrolled in these studies has changed, the framework by which the field describes that population has changed, and endpoint specification has followed neither. Patients with kidney disease no longer occupy the margin of cardiac intervention research, and the evidence assembled from their participation should account for both organ systems that brought them into the trial.
